# Non invasive evaluation of cardiomechanics in patients undergoing MitrClip procedure

**DOI:** 10.1186/1476-7120-11-13

**Published:** 2013-05-04

**Authors:** Fabio Guarracino, Baldassare Ferro, Rubia Baldassarri, Pietro Bertini, Francesco Forfori, Cristina Giannini, Vitantonio Di Bello, Anna S Petronio

**Affiliations:** 1 Department of Cardiothoracic Anesthesia and Intensive Care Medicine, Azienda Ospedaliero Universitaria Pisana, Via Paradisa 2, 56123, Italy; 2 Cardiothoracic and Vascular Department, Azienda Ospedaliero-Universitaria Pisana, Pisa, Italy; 3 Scuola Superiore di Studi Universitari e di Perfezionamento Sant’Anna, Piazza Santa Caterina, Pisa, Italy

**Keywords:** Mitraclip, Ventricular arterial coupling, Cardiomechanic

## Abstract

**Background:**

In the last recent years a new percutaneous procedure, the MitraClip, has been validated for the treatment of mitral regurgitation. MitraClip procedure is a promising alternative for patients unsuitable for surgery as it reduces the risk of death related to surgery ensuring a similar result. Few data are present in literature about the variation of hemodynamic parameters and ventricular coupling after Mitraclip implantation.

**Methods:**

Hemodynamic data of 18 patients enrolled for MitraClip procedure were retrospectively reviewed and analyzed. Echocardiographic measurements were obtained the day before the procedure (T0) and 21 **±** 3 days after the procedure (T1), including evaluation of Ejection Fraction, mitral valve regurgitation severity and mechanism, forward Stroke Volume, left atrial volume, estimated systolic pulmonary pressure, non invasive echocardiographic estimation of single beat ventricular elastance (Es(sb)), arterial elastance (Ea) measured as systolic pressure • 0.9/ Stroke Volume, ventricular arterial coupling (Ea/Es(sb) ratio). Data were expressed as median and interquartile range. Measures obtained before and after the procedure were compared using Wilcoxon non parametric test for paired samples.

**Results:**

Mitraclip procedure was effective in reducing regurgitation. We observed an amelioration of echocardiographic parameters with a reduction of estimated systolic pulmonary pressure (45 to 37,5 p = 0,0002) and left atrial volume (110 to 93 p = 0,0001). Despite a few cases decreasing in ejection fraction (37 to 35 p = 0,035), the maintained ventricular arterial coupling after the procedure (P = 0,67) was associated with an increasing in forward stroke volume (60,3 to 78 p = 0,05).

**Conclusion:**

MitraClip is effective in reducing mitral valve regurgitation and determines an amelioration of hemodynamic parameters with preservation of ventricular arterial coupling.

## Introduction

Mitral regurgitation (MR) is the second most frequent valve disease after aortic stenosis [1]. Mitral valve surgery is the standard of care for patients presenting symptomatic or asymptomatic MR with evidence of left ventricle (LV) dysfunction or dilation; mitral valve repair should be the preferred technique when it is expected to be durable [2]. However, many patients presenting a high surgical risk are treated with a strict titration of pharmacological therapy or resynchronization [3].

In recent years, a new percutaneous procedure using the MitraClip® has been validated for the treatment of mitral regurgitation. The positioning of a clip approximates leaflets at different locations mimicking Alfieri’s surgical suture and creating a double orifice mitral valve. For large orifices more clips can be used to effectively reduce regurgitant volume [4]. The Everest studies I and II demonstrated the safety and efficacy of the procedure [5]. Furthermore, Ussia et al. demonstrated an improvement in quality of life in high surgical risk patients undergoing the MitraClip procedure [6]. Therefore, the MitraClip procedure is considered to be a promising alternative for patients unsuitable for surgery ensuring a similar result.

One of the concerns when using this procedure in patients with advanced heart failure is that the acute correction of MR could further impair LV systolic performance, leading to a low or even lower cardiac output state [7,8].

Sunagawa demonstrated that the stroke volume produced by the heart is determined by the interaction between ventricular end-systolic elastance and arterial elastance [9]. Ventricular end-systolic elastance (Ees) is defined by the slope of the end-systolic pressure-volume relationship (ESPVR) and is a measure of the contractility of the left ventricle, which is not influenced by pre-load changes. Arterial elastance (Ea) is considered a good index of vascular load and is determined using the compliance, impedance and resistances of the arterial system. End-systolic pressure follows maximal ejection pressure because end-systole occurs as LV relaxation starts. Therefore, it can be approximated using the ratio of 90% of the systolic arterial pressure to Stroke volume (SV) [10]. Sagawa et al. demonstrated that the cardiovascular system is efficient and produces the maximal stroke work when the Ea/Ees ratio is 0.5-1 [11,12].

**Figure 1:**
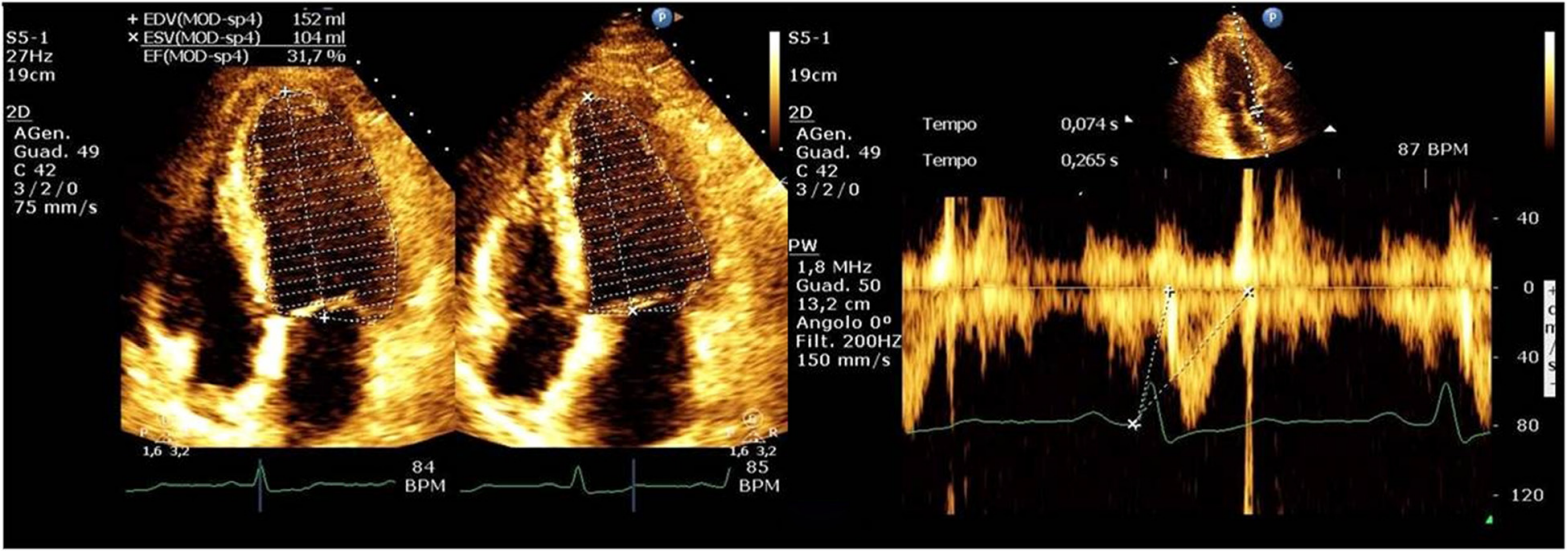
**Left ventricular end-systolic elastance was calculated by the single-beat method validated by Chen et al. **[14]**.** The echo figures display the evaluation of ejection fraction (left panel) and pre-ejection and ejection time (right panel) using aortic Doppler waveform. Normalized ventricular elastance at arterial end-diastole (End) was measured according to the formula: *E*_*es*(*sb*)_ = [*P*_*d*_ - (*E*_*Nd*(*est*)_ • *P*_*s*_ • 0.9)]/[*SV* • *E*_*Nd*(*est*)_], *E*_*Nd*(*avg*)_ = ∑ *ai* • *t*_*Nd*_ *i* = 0, *E*_*Nd*(*est*)_ = 0.0275 - 0.165 • *EF* + 0.3656 • (*P*_*d*_/*P*_*es*_) + 0.515 • *E*_*Nd*(*avg*)_ where a_i_ are (0.35695, -7.2266, 74.249, -307.39, 684.54. -856.92, 571.95, -159.1) for i = 1 to 7, respectively. The value for t_Nd_ was determined by the ratio of pre-ejection period (R wave to flow onset) to total systolic period (R wave to end-flow), with the time of onset and termination of flow defined Doppler. Systolic (Ps) and diastolic (Pd) blood pressure were invasively obtained.

Because measuring Ees varying preload and ventricular end-systolic pressures invasively is difficult in clinical practice, single-beat methods were developed and validated for obtaining the Ees value (Ees(SB)) [13]. In particular, the Ees [SB] can be evaluated by measuring the LV ejection fraction (EF), Stroke volume (SV), pre-ejection time and systolic time interval when coupled with the systolic and diastolic arterial pressures [14] (Figure 1).

For this reason, we aimed to evaluate the hemodynamic effects of the MitraClip procedure, with particular emphasis on cardiomechanics, by non invasive evaluation of ventriculo-arterial coupling.

## Methods

After receiving approval from the ethical committee for human biomedical investigation of Azienda Ospedaliero Universitaria Pisana, a retrospective analysis of prospectively collected data from patients who had undergone the MitraClip procedure was established. Patients with high surgical risk who met the echocardiographic criteria as defined by EVEREST II were included [15,16]. The parameters were measured by a single expert operator using a CX 50 ultrasound system (Philips, Bothell, WA 98041 USA) ) and a S5-1 Sector Array Transducer (Philips, Bothell, WA 98041 USA) the day before the procedure (T0) and after a period of 21 **±** 3 days after the procedure (T1) in all patients.

The analysis included the following: demographic data; an evaluation of the logistic EuroSCORE; mechanism of mitral regurgitation; evaluation of mitral regurgitation severity using a multiparametric quantitative and qualitative analysis as described in Table 1 and semiquantitive analysis using vena contracta; the mean transmitral gradient; left ventricular end-diastolic and end-systolic volumes; the echocardiographic estimation of systolic pulmonary arterial pressure; ejection fraction; the non-invasive estimation of Ees(SB) elastance (Figure 1) [14]; arterial elastance (Ea) assessed as the ratio between 0,9* systolic pressure to Stroke Volume; and the Ea/Ees(SB) ratio. All data were analysed offline.

**Table 1:** **Echocardiographic criteria used to define severity of mitral regurgitation based on multi parametric quantitative and qualitative analysis as suggested by american Society of Echocardiography Reccomendations **[16]

**Mitral Regurgitation Criteria**				
**Grade**	**Mild 1+**	**Moderate 2+**	**Moderate to severe 3+**	**Severe 4+**
**Color flow Doppler**	<4 cm2 or <10 cm2 LA area	4-6 cm2 or 10-30% of left LA area	6 to <8 cm2 or 30% to <40% of LA area	>8 cm2 or >40% of LA area
**Regurgitant Volume (ml/beat)**	<30	30-44	45-59	>60
**Regurgitant Fraction (%)**	<30	30-39	40-49	>50
**Pulmonary Vein flow**	Systolic Dominant	Diastolic Dominant	All Diastolic	Systolic Reversal

### Statistical analysis

The data are presented as the mean **±** SD or the median **±** interquartile range. A non-parametric Wilcoxon test for paired samples was used because of the small number of patients. Results were considered significant when p < 0,05. Correlations between the Ees(SB) and the ejection fraction were evaluated using the Spearman coefficient.

## Results

We reviewed forty cases of the MitraClip procedure performed over two years (from June 2010 to June 2012). We found complete echocardiographic data for 18 patients (11 males and 7 females) with a mean age of 73 **±** 1 years, who were then considered in the analysis.

**Figure 2:**
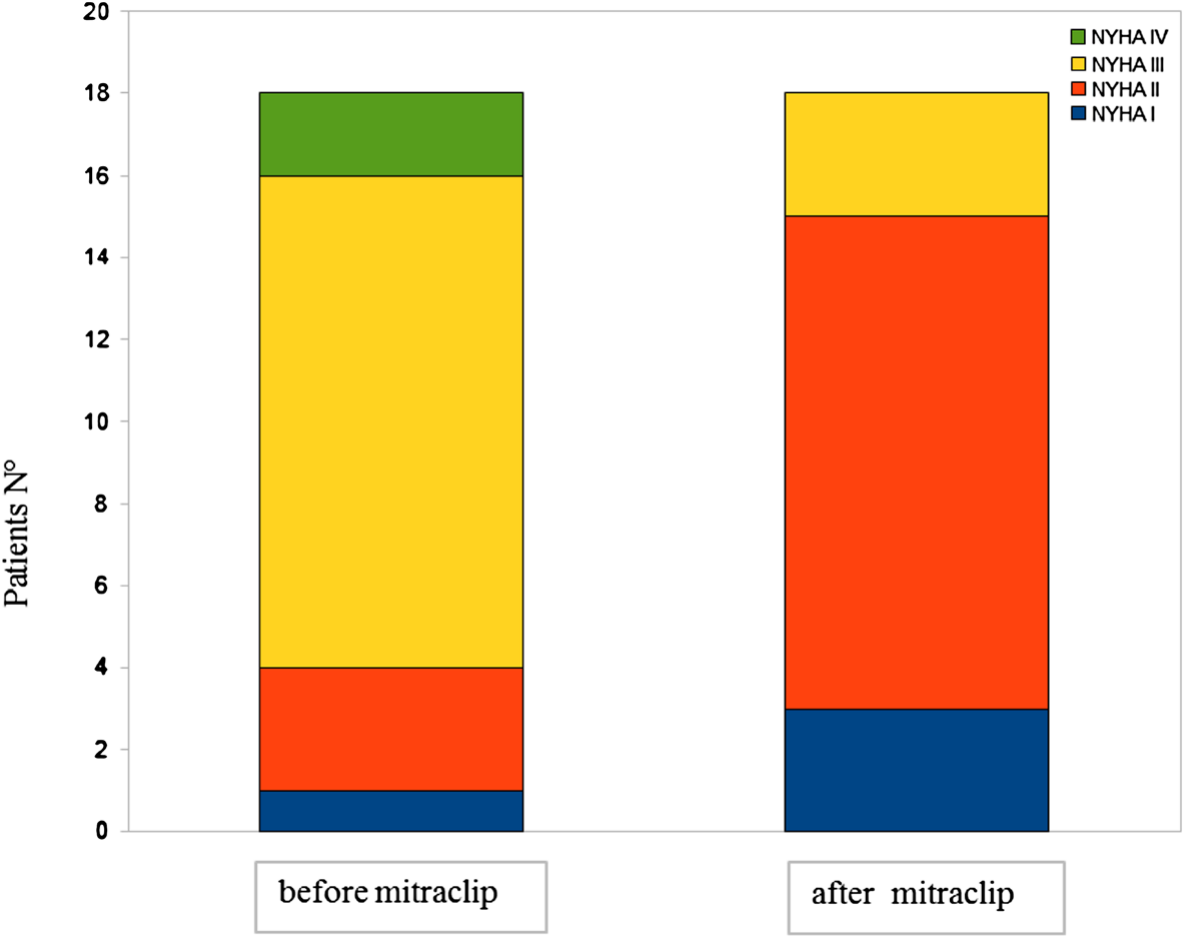
**Reduction of Mitral Valve regurgitation assessed with echocardiographic method in awake patients before MitraClip positioning (T0) and after 21 ± 3 days from procedure.**

**Figure 3:**
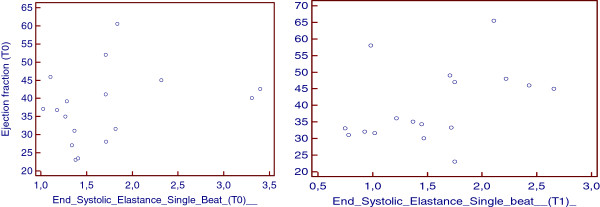
Lack of correlation between Single Beat End Systolic Elastance (EesSb) and Ejection fraction before (T0) (left) and after 21 ± 3 days (T1) from MitraClip positioning (right).

The patients presented a logistic EuroSCORE of 30,8 ±25,2. All patients presented a diagnosis of functional MV regurgitation. The results are summarized in Table 2. The MitraClip procedure effectively reduced MV regurgitation (Figure 2) without causing a significant increase in the mean transmitral diastolic gradient. We observed a reduction in the left atrial volume, estimated systolic pulmonary pressure and end-systolic and end-diastolic LV volumes. Despite a decrease in the ejection fraction, the patients showed an increase in forward stroke volume. We did not observe variations in the end-systolic single-beat elastance or arterial elastance or the Ea/Ees(Sb) ratio. There was no correlation between the Ees(SB) and the ejection fraction before (Spearman coefficient =0,2) or after the procedure (Spearman coefficient = 0,3) (Figure 3).

**Table 2:** Echocardiographic parameters assessed in awake patients measured the day before MitraClip positioning and at discharge from hospital

	**Before MitraClip procedure (T0)**	**After MitraClip procedure (T1)**	**p**
MV regurgitation Jet area (cm2)	11,8	6,3	P < 0,0001
	8,3 to 14,575	4,7 to 8	
Vena contracta (cm)	0,8	0,4	P < 0,0001
	0,65 to 0,9	0,3 to 0,42	
Left atrial volume (ml)	110,5	93,5	P < 0,0001
	88 to 122	66 to 105	
Estimated Systolic Pulmonary Pressure (mmhg)	45	37,5	P = 0,0002
	35 to 50	30 to 45	
Ejection Fraction (%)	37	35	P = 0,035
	30,25 to 43,125	31,9 to 47,25	
Mean Transmitralic Gradient (mmHg)	2,95	3	P = 0,15
	2 to 4	3 to 4	
End Systolic Volume (ml)	106	97	P = 0,001
	55,5 to 129,75	40,75 to 120,5	
End Diastolic Volume (ml)	159,5	151	P < 0,0001
	113 to 197	89 to 180	
Forward stroke volume (ml)	60,3	78	P = 0,05
	49,85 to 73,1	54,5 to 86	
Arterial elastance (mmhg/ml)	1,225	1,22	P = 0,30
	0,937 to 1,44	0,794 to 1,44	
End Ejection Systolic Elastance (SB) (mmHg/ml)	1,43	1,46	P = 0,37
	1,286 to 1,82	1,02 to 1,75	
Ventriculo-Arterial Coupling (Ea/Es)	0,765	0,765	P = 0,67
	0,654 to 0,839	0,689 to 0,839	

Data are expressed as median **±** IR. P significative < 0,05.

**Figure 4:**
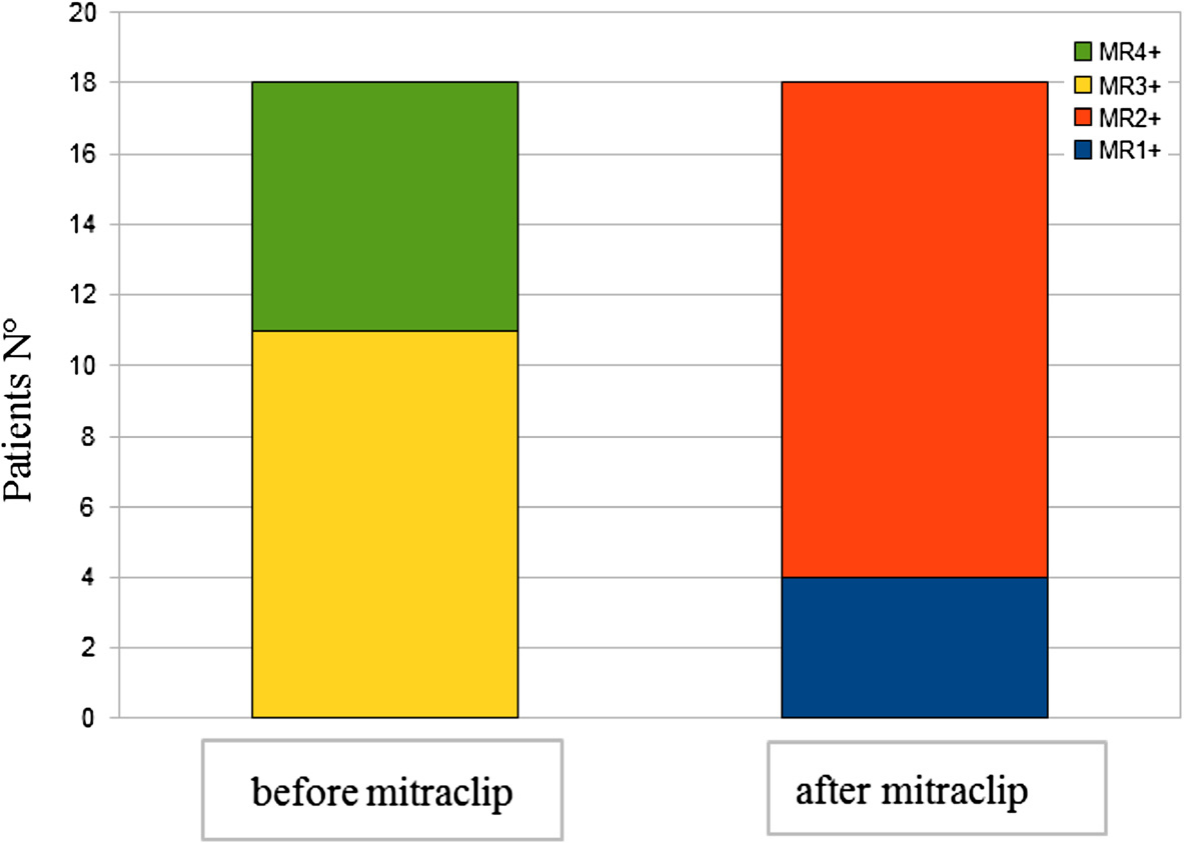
NYHA class status assessed before MitraClip positioning (T0) and after 21 ± 3 days (T1) from procedure.

Following the MitraClip procedure, the NYHA class was significantly reduced in our patients (p = 0,0001) (Figure 4).

## Discussion

The main finding of this study was that the reduction of mitral regurgitation with MitraClip procedure is associated with preserved ventricular end systolic elastance (single beat) not correlated with the reduction of EF, and unchanged ventriculo-arterial coupling defined as Ea/Ees(SB) at an early follow-up.

A percutaneous edge-to-edge procedure may be considered in patients with symptomatic severe MR who fulfill the echo criteria of eligibility, are judged inoperable or at high surgical risk by a ‘heart team’, and have a life expectancy greater than 1 year (recommendation class IIb, level of evidence C) [2]. The impact of percutaneous therapy allows the treatment of patients for whom the only alternative is amelioration using pharmacological therapy without a significant improvement in the duration or quality of life due to unacceptable surgical risk.

Our study confirms the hemodynamic effects of the MitraClip procedure for patients with severe mitral regurgitation. All patients presented a significant reduction of mitral regurgitation at the time of control (T1). The reduction of MV insufficiency was associated with lowering of PAPs, left ventricular and atrial volumes and amelioration of the NYHA class status.

We hypothesize that the observed reduction of the ejection fraction could not be an index of decreased systolic function, rather a consequence of a reduced preload, as evidenced by the reduction of the left atrial and ventricular volumes. Left ventricular unloading is considered one of the most important determinants of the reduction of clinical symptoms in patients after correction of mitral regurgitation by any technique (19). In the MitraClip procedure closing the MV regurgitant orifice lowers the regurgitation volume and the left ventricular and diastolic volumes during the next diastole.

The analysis of ventriculo-arterial coupling in this subset of patients produced interesting results.

Siegel et al. have shown that successful MV repair with the MitraClip system results in an immediate improvement in FSV, CO, and LV loading conditions and an acute reduction of the systemic vascular resistance [17].

A post-hoc analysis has demonstrated that the hemodynamic benefit of the percutaneous procedure is higher in a particular subset of patients with a low CI and a high left-sided filling pressure and PAP [18].

Ken-ichi Imasaka et al. demonstrated that the early surgical repair of organic mitral valve regurgitation influenced ventricular coupling in patients with preserved and impaired LV function. Measuring ventricular and arterial coupling before and 1 month after surgery, they showed that Ea increased after the procedure in all patients, but only patients with preserved function increased their contractility to rebalance ventricular coupling [19].

Most of our patients presented impaired function with a maintained optimal Ea/Ees(SB) ratio before the procedure. We demonstrated that contractility was preserved in all patients and that Ea was not increased after the procedure. As a consequence, the lack of variation in the Ea/Ees(SB) ratio is the expression of maintained ventriculo-arterial coupling and ventricular performance after the percutaneous closure of the defect. Considering stroke volume as a function of ventriculo-arterial coupling, we can deduce that, despite reducing the EF, the combination of the restoration of normal blood ejection and the preserved Ea/Ees ratio increases the forward “effective” stroke volume after the MitraClip procedure.

Our findings are supported by the recently published data by Gaemperli et al. who investigated the acute behavior of pressure volume relationships in patients undergoing Mitraclip procedure. Based on the invasive measurements obtained with conductance catheters during the intervention they found no acute variations of PV loop area and increased CI following MV repair [20].

Our study shows limitations due to its retrospective design and the small number of patients analyzed. Our results have been obtained in a non-invasive manner and we cannot exclude differences if data were obtained invasively monitoring ventricular and vascular load determinants, although recent evidence seems to support our results [20]. However, this study is the first to non invasively describe midterm preserved ventricular performance and the low impact of MitraClip repair of MV regurgitation on LV afterload (Ea) in patients with impaired ventricular function. Larger prospective studies are needed to confirm these results focusing on relation between preservation of cardiomechanic parameters and clinical status during follow up in patients undergoing MitraClip procedure.

## Consent

Written informed consent was obtained from the patients for publication of this report and any accompanying images.

Key message:

• MitraClip is an effective procedure in reducing mitral valve regurgitation in high surgical risk patients.

• MitraClip procedure has favorable hemodynamic impact. The reduction of Mitral regurgitation is associated with preserved Ea/Ees(sb) ratio and increased forward stroke volume.

• Non invasive evaluation of cardiomechanics is feasible and may be useful in patients undergoing MitraClip procedure.

## Competing interests

None of the authors have any financial competing interests.

## Authors’ contributions

FG: participated in the design of the study, wrote and reviewed the manuscript. FB: collected data, wrote and reviewed the manuscript. BR: collected data and reviewed the manuscript. BP: has been involved in drafting the manuscript and revising it critically for important intellectual content. GC: collected data and reviewed the manuscript. DBV: has been involved in critically revising the final manuscript. PAS: has been involved in drafting the manuscript and revising it critically for important intellectual content. All authors approved the final version of manuscript.
